# Attitudes and Perception of the *REFLECT* Communication Curriculum for Clinical Oncology Graduate Medical Education

**DOI:** 10.1007/s13187-023-02333-5

**Published:** 2023-06-22

**Authors:** Brady S. Laughlin, Natalie Langley, Samir H. Patel, Katherine Kough, Brenda Ernst, Jonathan B. Ashman, William G. Rule, Tamara Z. Vern-Gross

**Affiliations:** 1 Department of Radiation Oncology, Mayo Clinic, 5881 E Mayo Blvd., Phoenix, AZ 85054 USA; 2 Department of Laboratory Medicine and Pathology, Mayo Clinic, Phoenix, AZ USA; 3 Department of Humanities, Mayo Clinic, Phoenix, AZ USA; 4 Department of Hematology/Oncology, Mayo Clinic, Phoenix, AZ USA

**Keywords:** Communication curriculum, Reflective writing, Oncology training

## Abstract

Communication and interpersonal skills are essential components of oncology patient care. The REFLECT (*R*espect, *E*mpathy, *F*acilitate Effective Communication, *L*isten, *E*licit Information, *C*ompassion, and *T*each Others) curriculum is a novel framework to improve and refine physician/patient interactions for oncology graduate medical trainees. We seek to evaluate the attitudes and perceptions of the REFLECT communication curriculum among oncology trainees. Seven-question and 8-question Likert scale surveys (1 = not beneficial and 5 = beneficial) were distributed to resident/fellow participants and faculty mentors, respectively. Questions asked trainees and faculty about their perceptions of improvement in communication, handling of stressful situations, the value of the curriculum, and overall impression of the curriculum. Descriptive statistics determined the survey’s baseline characteristics and response rates. Kruskal–Wallis rank sum tests were used to compare the distribution of continuous variables. Thirteen resident/fellow participants completed the participant survey. Six (43.6%) Radiation Oncology trainees and 7 (58.3%) Hematology/Oncology fellows completed the trainee survey. Eight (88.9%) Radiation Oncologists and 1 (11.1%) Medical Oncologist completed the observer survey. Faculty and trainees generally felt that the curriculum increased communication skills. Faculty responded more favorably to the program’s impact on communication skills (median 5.0 vs. 4.0, *p* = 0.008). Faculty were more assertive about the curriculum’s capabilities to improve a learner’s ability to handle stressful situations (median 5.0 vs. 4.0, *p* = 0.003). Additionally, faculty had a more favorable overall impression of the REFLECT curriculum than the residents/fellows (median 5.0 vs. 4.0, *p* < 0.001). Radiation Oncology residents felt more strongly that the curriculum enhanced their ability to handle stressful topics, compared to Heme/Onc fellows (median 4.5 vs. 3.0, range 1–5, *p* = 0.379). Radiation Oncology trainees felt more consistently that the workshops improved their communication skills, compared to Heme/Onc fellows (median 4.5 vs. 3.5, range 1–5, *p* = 0.410). The overall impression between Rad Onc resident and Heme/Onc fellows was similar (median 4.0, *p* = 0.586). Conclusions: Overall, the REFLECT curriculum enhanced communication skills of trainees. Oncology trainees and faculty physicians feel that the curriculum was beneficial. As interactive skills and communication is critical to build positive interactions, further work is needed to improve the REFLECT curriculum.

## Introduction

As physicians care for patients undergoing cancer treatment, oncologists play a multifaceted role for patients and their families [1]. While communication is a crucial skill in oncology and palliative care, there are layered complexities when working with people fighting an illness such as cancer, threatening their livelihoods and survival. Patients with cancer are often in their most vulnerable state; they report unmet needs regarding staging, prognosis, management options, the intent of therapy, and toxicity [2]. Since patients want to discuss these crucial issues, physicians need to identify the timing to confidently discuss these critical topics [3].

Oncologists’ responsibilities include communicating about diagnosis and prognosis, navigating patient emotions, determining patient/family verbal and mental capacity, and self-management [1, 4]. Eight critical communication skills for oncology professionals were identified by Kissane et al. as follows: (1) breaking bad news; (2) discussing prognosis and risk; (3) shared decision making; (4) responding to emotions; (5) handling recurrence; (6) transitioning to palliative or end of life care; (7) running a family meeting; (8) discussing death and dying [5]. If not adequately trained to focus on interpersonal communication and affect, physicians may lack nuanced skills to handle specific clinical encounters [6–8]. Strong communication skills benefit clinicians in many ways: patients are more likely to adhere to treatment, experience reduced anxiety, report higher satisfaction, have fewer malpractice claims, and have better overall outcomes [9]. Reflective practices also enhance a physician’s capability to evaluate patients and improve empathy and communication skills [10]. Furthermore, improving communication skills and reflection can reduce stress and burnout among healthcare professionals [11, 12].

**Fig. 1:**
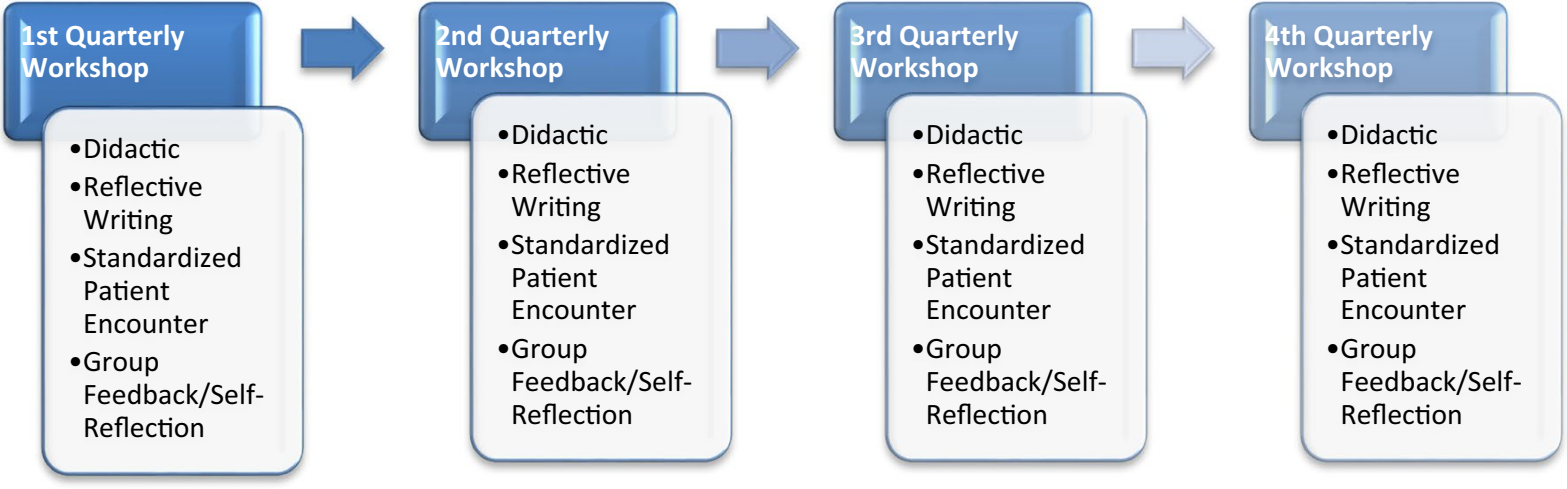
REFLECT curriculum design

The REFLECT curriculum was implemented in 2018 at a single institution as a novel, core communication curriculum initially for Radiation Oncology residents and then expanded to include Hematology/Oncology fellows. REFLECT means *R*espect, *E*mpathy, *F*acilitate Effective Communication, *L*isten, *E*licit Information, *C*ompassion, and *T*each Others. The REFLECT curriculum uses reflective processes to strengthen medical education and professional identity development [13, 14]. The curriculum consists of reflective narrative writing, evaluation, and comprehensive quarterly workshops with concurrent curriculum participation throughout the residency or fellowship training. Each year, this comprehensive course consists of quarterly (4-h) workshops comprising assigned reading, knowledge assessments, didactic lectures, expert guest lecturers, standardized patient (SP) simulations, role-playing, patient/expert panels, coaching, reflective writing, and debriefing/feedback sessions on relevant issues (Fig. 1). Trainees were taught the SECURE framework (Set the Stage, Elicit Information, Convey Information, Understand the Patient’s Perspective, Respond to Emotions, End the Encounter) American Academy of Hospice and Palliative Medicine (AAHPM) in their approach to SP simulations [15]. This study was developed to assess faculty and trainee perceptions of the REFLECT curriculum, understand its impact, and identify areas of improvement.

## Materials/Methods

Surveys were developed to obtain perceptions of the REFLECT curriculum from trainee (Radiation Oncology and Medical Oncology) participants and faculty mentors. Surveys were designed so that participation was anonymous. Additionally, participation was voluntary and non-incentivized. The surveys asked pertinent questions regarding perceptions of the REFLECT curriculum. Surveys were sent out to participants and faculty who participated in at least 1 REFLECT curriculum workshop.

Seven-question and 8-question Likert scale surveys (1 = not beneficial and 5 = beneficial) were developed and distributed to oncology residents, fellows, and faculty mentors. Both surveys contained questions asking respondents to provide constructive feedback regarding the curriculum. Questions assessed trainee and faculty perceptions of improvement in communication, handling of stressful situations, the value of the curriculum, and overall impression of the curriculum. Faculty surveys also included questions regarding their assessment of the impact on trainees in the curriculum and whether the curriculum would have benefited their training. Descriptive statistics determined the survey’s baseline characteristics and response rates. Kruskal–Wallis rank sum tests were used to compare the distribution of continuous variables.

## Results

Thirteen resident/fellows completed the participant survey. Six (46.1%) Radiation Oncology trainees and 7 (58.3%) Hematology/Oncology fellows completed the survey (Table 1). Nine faculty members, including 8 (89%) Radiation Oncologists and 1 (11%) Medical Oncologist, completed the faculty survey. The response rate was 13/16 trainees (81.3%) and 9/13 physicians (69.2%). Table 2 demonstrates the responses of the residents and fellows, and Table 3 highlights the responses provided by faculty.

**Table 1:** REFLECT Curriculum Workshop topics

Topic
Delivering Bad News
The Challenging Patient
Prognostication for the Incurable Patient
The Adolescent Patient
Managing Conflict with Colleagues
The Patient Facing Mixed Messages
Opioid Diversion
Honoring Cultural and Religious Preferences
Communication and Delivery of Bad News
The Angry Patient
The Gray Line — Defining Boundaries with the Physician and Patient
Provider “Burn Out” or “Mental Fog”

**Table 2:** Trainee/fellow survey

What is your current/prior role?	Radiation oncology 6 (41.7%)	Hematology/oncology 7 (58.3%)			
Likert Scale	1	2	3	4	5
Do you feel the REFLECT curriculum improved your communication skills?	1 (7.7%)	2 (15.4%)	2 (15.4%)	6 (46.2%)	2 (15.4%)
Do you feel the topics addressed within the REFLECT curriculum enhanced your ability to handle stressful situations?	1 (7.7%)	2 (15.4%)	3(23.1%)	4 (30.8%)	3(23.1%)
Do you feel the topics addressed within the REFLECT curriculum enhanced your ability to handle certain topics as a clinician?	1 (7.7%)	2 (15.4%)	1 (7.7%)	7 (53.8%)	2 (15.4%)
Do you believe this type of curriculum would have been essential to your training?	2 (15.4%)	3 (23.2%)	4 (30.8%)	2 (15.4%)	2 (15.4%)
Do you feel the content of these workshops enhanced your communication skillsets?	1 (7.7%)	2 (15.4%)	2 (15.4%)	4 (30.8%)	3 (23.2%)
What is your overall impression of the REFLECT curriculum?	2 (15.4%)	1 (7.6%)	1 (7.6%)	7 (53.8%)	2 (15.4%)
Do you feel that the topics in this curriculum are relevant to the field of oncology and implementation in clinical care?	0 (0)	1 (7.6%)	3 (23.1%)	6 (46.2%)	3 (23.1%)

**Table 3:** Faculty survey (1 = not beneficial; 5 = beneficial)

What is your current/prior role?	Radiation Oncology Physician	Hematology/Oncology Physician			
	8 (88.9%)	1 (11.1%)			
Have you observed residents or fellows participate in the REFLECT curriculum?	Yes	No			
	9 (100%)	0			
Likert Scale	1	2	3	4	5
Do you feel the REFLECT curriculum improved the resident/fellow's communication skills?	0	0	0	3 (33.3%)	6 (66.7%)
Do you feel the topics addressed within the REFLECT curriculum enhanced the trainees’ ability to handle stressful situations?	0	0	0	1 (11.1%)	8 (88.9%)
Do you feel the topics addressed within the REFLECT curriculum enhanced your ability to handle certain topics as a clinician?	0	0	0	4 (44.4%)	5 (55.6%)
Do you believe this type of curriculum would have been essential if incorporated into your own training?	0	0	0	3 (33.3%)	6 (66.7%)
Do you feel the content of these workshops enhanced communication skillsets of trainees?	0	0	0	1 (11.1%)	8 (88.9%)
Do you feel the content of this workshop enhanced your own communication skills? (1 = not beneficial, 5 = beneficial)	0	0	0	3 (33.3%)	6 (66.7%)
What is your overall impression of the REFLECT curriculum?	0	0	0	0	9 (100%)
Do you feel that the topics in this curriculum are relevant to the field of oncology and implementation in clinical care?	0	0	0	1 (11.1%)	8 (88.9%)

In general, faculty responded more favorably to the beneficial aspects of the curriculum. Faculty and trainees generally felt the curriculum increased communication skills (median 5.0 vs. 4.0, mean 5.0 vs. 3.5, *p* = 0.008). Faculty were more assertive about the curriculum's capabilities to improve an Oncology trainee’s ability to handle situations (median 5.0 vs. 4.0, mean 4.9 vs. 3.5, *p* = 0.003). Additionally, residents did not have a strongly favorable impression of the REFLECT curriculum (median 5.0 vs. 4.0, mean 5.0 vs. 3.5, *p* < 0.001). Similarly, faculty felt more strongly about the relevance of the curriculum to the field of oncology and clinical care implementation (median 5.0 vs. 4.0, mean 4.9 vs. 3.8, *p* = 0.003). The range for all faculty responses was 4.5–5.0. On the other hand, the range for trainee responses was 1–5.

When comparing Medical Oncology fellows and Radiation Oncology residents, there were no differences in perceptions of the curriculum. Radiation Oncology residents and medical oncology fellows felt similarly about the impact on communication skills (median 4.0 vs. 4.0, mean 3.5 vs. 3.4, *p* = 0.597). Radiation Oncology residents felt more assertive about the curriculum helping improve their ability to handle certain situations (median 4.5 vs. 3.0, mean 3.7 vs. 3.3, *p* = 0.379). Both groups had a similar impression of the curriculum, with medical oncology fellows feeling it was slightly non-beneficial (median 3.0 vs. 3.0, mean 2.7 vs. 3.2, *p* = 0.609).

Faculty felt stronger about the curriculum’s capabilities to improve their ability to handle stressful situations (median 5.0 vs. 4.0, mean 4.9 vs. 3.5, *p* = 0.003). Additionally, the residents did not have as strongly favorable impression of the REFLECT curriculum (median 5.0 vs. 4.0, mean 5.0 vs. 3.5, *p* < 0.001).

In general, trainees felt the curriculum improved their communication skills (mean = 3.5, range 1–5). Most also felt the workshop was beneficial in preparing them to handle specific topics (69.2%). Seven participants (54%) felt that the curriculum enhanced their communication. However, 23% of trainees felt it was not beneficial for their training. The overall impression was positive, with 77% of participants rating the curriculum as beneficial. Faculty had a significantly more positive perception of the REFLECT than trainees (*p* < 0.05).

Trainee feedback noted that the curriculum might not be as beneficial for Medical Oncology fellows. One trainee commented, “Communication workshops are always very valuable. However, I believe this can be part of the curriculum for first-year residents and/or fellows and does not need to extend into additional training years.” The notion that participation is reduced to only occur in the early years of training was mentioned in six of nine comments (67%). Another comment (11%) noted that workshops could be shorter. Two comments noted that the curriculum was helpful for oncology trainees (22%). On the other hand, most faculty comments provided praise for the curriculum (75%).

## Discussion

The American Society of Clinical Oncology (ASCO) has drafted guidelines to enhance communication between patients and physicians [16]. ASCO revealed that many oncologists have deficiencies in communicating complex information and assisting patients and their loved ones in decision-making [16]. Therefore, ASCO recommended training oncologists to acquire and build strong communication and interpersonal skills. Research has demonstrated that well-designed communication training programs can improve a clinician’s communication and the patient experience [17, 18].

The REFLECT curriculum sought to improve clinical communication skills and reflective processes for Radiation Oncology residents and Medical Oncology fellows. The REFLECT curriculum created a framework for multiple skills to be incorporated into memory and implemented into clinical practice, building upon the trainee’s unique traits and abilities. Communication courses standardly incorporated into improving the skillsets of medical oncologists often focus on challenging conversations such as prognostication and delivering bad news. For instance, Oncotalk is a 2-day course in which learners of all skill levels participate in simulated sessions of clinical scenarios to enhance interactions and communication skillsets [19]. While Oncotalk is successful in enhancing skill sets, it has several limitations. As Oncotalk is not concurrent with any formal residency or fellowship curriculum, it is disconnected from situational learning. On the other hand, the REFLECT curriculum is longitudinal and concurrent with the residency/fellowship curriculum.

This is one of several communication curriculums employed for Radiation Oncology residents and Medical Oncology fellows in the USA. Christensen et al. reported positive improvement in observable communication skills among radiation oncology residents who participated in serious illness communication training [20]. Cannone and colleagues developed an eight-module course on communication in oncology practice, delivered over 2 months, to a multi-specialty group consisting of palliative medicine fellows, medical oncology fellows, and Radiation Oncology residents [21]. Similar to our study, there was perceived improvement in communication skills and comfort across multiple domains [21]. Levy et al. undertook a qualitative approach to identify and reflect upon areas of improvement in communication skills training for medical oncology fellows and Radiation Oncology trainees of the Royal Australian and New Zealand College of Radiologists (FRO RANZCR) [22]. For eight sessions, they identified enablers and barriers to effective communication, written communication, communicating bad news, and multidisciplinary team meeting collaboration as essential themes.

The survey demonstrated that Oncology trainees (Radiation Oncology and Medical Oncology) felt the REFLECT curriculum positively impacted their communication skills and ability to handle specific clinical scenarios. However, this did not correspond entirely to the belief that the curriculum benefited their training. On the other hand, faculty physicians felt the curriculum improved communication skills. A significant difference in the impact of the curriculum on practice may stem from experience gained over time by faculty. Therefore, future research should assess retrospective perceptions and attitudes about the REFLECT curriculum.

The current study is not without limitations. A small cohort of trainee participants and faculty observers limits this current study. Additionally, the survey could have been more comprehensive regarding their perception of specific workshops and topics. However, a more comprehensive survey was not chosen as fewer responses would be anticipated due to survey fatigue. A Likert scale of 1–5 was chosen to simplify the decision-making for trainees and faculty regarding their perceptions of the curriculum. A larger scale could be considered to understand trainee perceptions better. Although feedback was gathered, feedback was solely focused on reducing the total time of the workshops and oncology fellow involvement. Feedback could have been gathered more comprehensively to ascertain how to enhance the various components of the REFLECT curriculum sessions, including lectures, group discussions, and standardized patient simulations.

This study has helped identify the limitations of the REFLECT curriculum. A significant component of the curriculum is enhancing reflective processes in oncology trainees to increase self-awareness while facing tremendously uncertain situations. Oncology fellows may perceive the communication curriculum as not a good use of their time because they are further into their medical education. Given trainees’ perception of the curriculum, this exposes a gap the curriculum seeks to improve. However, further evaluation may reveal that these trainees feel “de-skilled” by the expectation to perform communication skills for which they feel already competent [23]. Future directions for the REFLECT curriculum will emphasize reflective processes while enhancing faculty involvement. Additionally, the curriculum will seek to adapt practices adopted in other successfully implemented curriculum such as one published by Christensen et al. [20]. In conclusion, we plan to identify ways to maximize the improvement of communication skills and positive perception of the program. Additionally, the curriculum will work to assess communication skills formally.

## Data Availability

The datasets generated during the current study are not publicly available due to restricted access to institutional repository but can be available from the corresponding author on reasonable request.
